# LINC01614 Promotes Colorectal Cancer Cell Growth and Migration by Regulating miR-217-5p/HMGA1 Axis

**DOI:** 10.1155/2023/6833987

**Published:** 2023-05-31

**Authors:** Jiwei Jia, Pei Guo, Li Zhang, Wenqing Kong, Fangfang Wang

**Affiliations:** ^1^Department of Radiation Oncology, Yantai Yuhuangding Hospital, 20 Yuhuangding East Road, Yantai, Shandong 264000, China; ^2^Department of Pathology, Yantai Yuhuangding Hospital, 20 Yuhuangding East Road, Yantai, Shandong 264000, China; ^3^Central Ward Operating Room, Yantai Yuhuangding Hospital, 20 Yuhuangding East Road, Yantai, Shandong 264000, China; ^4^Outpatient Operating Room, Yantai Yuhuangding Hospital, 20 Yuhuangding East Road, Yantai, Shandong 264000, China

## Abstract

Colorectal cancer (CRC) substantially contributes to cancer-related deaths worldwide. Recently, a long non-coding RNA (lncRNA), LINC01614, has emerged as a vital gene regulator in cancer progression. Yet, how LINC01614 affects CRC progression remains enigmatic. Here, we defined LINC01614 expression in CRC, investigated the performance of CRC cells lacking LINC01614, and elucidated the underpinning mechanism. We observed that LINC01614 was upregulated in both CRC tissues and cell lines. LINC01614 knockdown repressed the proliferation and metastasis capacity of CRC cell lines. Consistently, an *in vivo* LINC01614 deficiency model exhibited slow tumor growth rate. Moreover, luciferase reporter assay, RNA pull-down, and immunoprecipitation confirmed that LINC01614 targeted miR-217-5p. LINC01614 knockdown reduced the expression of HMGA1 and N-cadherin, while increasing that of E-cadherin, resulting in decreased viability, proliferation, migration, and invasion capacity of CRC cells. Our results demonstrate that LINC01614 regulates CRC progression by modulating the miR-217-5p/HMGA1 axis, thus holding great potential as a prognostic biomarker for CRC diagnosis and treatment.

## 1. Introduction

Colorectal cancer (CRC) accounts for 10% of new cancer cases globally, with one to two million newly diagnosed patients each year [1]. And it ranks third in incidence and second in cancer mortality with 700,000 deaths annually [2]. Approximately, 25% of CRC cases are familial [3]. Aging is another major risk factor of developing CRC, and the probability of developing CRC increases significantly in people aged 50 years older [4]. Recently, non-coding RNAs (ncRNAs) have been found to be essential for the pathogenesis of cancers [5]. Long non-coding RNAs (lncRNAs) are a type of ncRNA and have emerged as a critical gene regulator in cancer pathogenesis and progression [6, 7].

LncRNAs are important gene regulators in CRC. LncRNA DLEU1 contributes to CRC progression by activating KPNA3 [8]. Overexpression of lncRNA DANCR is associated with tumor progression and poor prognosis [9]. Similarly, lncRNA MALAT1 is upregulated in CRC, leading to poor prognosis in patients [10]. A circulating lncRNA, SNHG11, has been reported as a novel biomarker for the diagnosis of early CRC [11]. Moreover, lncRNAs have been proposed as a linker between RNA regulation and CRC progression [12].

Long intergenic non-coding RNA 01614 (LINC01614) is located on chromosome 2q35, encoding a lncRNA of 2904 nucleotides (nt) [13]. LINC01614 dysregulation is associated with cancer development. For instance, LINC01614 is upregulated in breast cancer, which is associated with poor prognosis [14]. Similarly, upregulation of LINC01614 has been proposed as a prognostic indicator of survival in non-small cell lung cancer (NSCLC) [15], and LINC01614 knockdown inhibits lung adenocarcinoma progression [13]. Moreover, LINC01614 is upregulated in glioma [16]. However, the role of LINC01614 in CRC has not yet been explored. Elucidation of the function and regulation mechanism of LINC01614 can help design new therapeutic strategies for CRC treatment and provide a new biomarker for CRC diagnosis. Here, using a combination of cell biology and molecular techniques, we dissected the role of LINC01614 in the pathogenesis and progression of CRC.

## 2. Materials and Methods

### 2.1. Cell Culture and Transfection

NCM460 cells were cultured in RPMI-1640 (Thermo Fisher Scientific, USA), SW620 cells in DMEM (Thermo Fisher Scientific, USA), HCT116 and HT-29 cells in McCoy's 5a Modified Medium (Thermo Fisher Scientific, USA), LOVO cells in ATCC-formulated F-12K medium (ATCC, USA), and SW480 cells in ATCC-formulated Leibovitz's L-15 medium (ATCC, USA) under standard laboratory conditions in an incubator at 37°C with 5% CO_2_. All the media were supplemented with 10% fetal bovine serum (FBS) and 1% penicillin-streptomycin (Gibco-Thermo Fisher Scientific, USA). Cells were grown to 70–90% confluence at transfection. Lipofectamine reagent 2000 (Invitrogen, USA) and transfection constructs were diluted separately in the Opti-MEM^®^ Medium (Thermofisher, USA) and mixed at 1 : 1 followed by incubation for 5 minutes at room temperature. The resulting lipid complex was incubated with the cells at 37°C for 48 hours.

### 2.2. Collection of Clinical Samples

Thirty-six paired CRC and normal tissue samples were collected from patients by surgery with written informed consent at Yantai Yuhuangding Hospital from May 2017 to October 2020. CRC samples were confirmed by histopathological examinations. None of the patients were treated with chemotherapy or radiotherapy before the surgery. Paired adjacent non-tumor tissues were dissected from a healthy area 5 cm away from the tumor edge and were confirmed to be negative for tumor cells. The samples were frozen at −80°C immediately after collection. All experimental procedures were approved by the ethical committee for human experimentation at Yantai Yuhuangding Hospital and were performed in accordance with the declaration of Helsinki.

### 2.3. Cell Viability Assay

The cell counting kit 8 assay (CCK-8) was used to determine the cell viability. Briefly, approximately 1000 cells/well were seeded into 96 well cell culture plates and cultured overnight. After treatments, cells were incubated for 24, 48, and 72 hours. Subsequently, 10 *μ*L of CCK-8 (Dojindo, Japan) was added to each well, followed by incubation for 1 hour at 37°C. Finally, the absorbance at 450 nm wavelength was measured using the Elx800 absorbance reader (BioTek Instruments).

### 2.4. Colony Formation Assay

The proliferation of CRC cells was examined by the colony formation assay. Approximately 500 cells/well with or without transfection were seeded into 24-well plates and cultured for 10 days under standard laboratory conditions. Further, the cells were fixed with 100% methanol (Sigma, USA), stained with 0.1% crystal violet solution (Solarbio, China), and manually counted under a laboratory microscope (Olympus, Japan).

### 2.5. Flow Cytometry Analysis

Annexin V-FITC/PI Apoptosis Detection Reagent (Invitrogen, USA) was used to calculate cell apoptosis ability following the supplier's protocol. The apoptotic cells were calculated using a Beckman Coulter FACS flow cytometer (Beckman Coulter, USA) coupled with the FlowJo software system (Ashland, USA).

### 2.6. Western Blot

Cells were lyzed in ice-cold radioimmunoprecipitation assay (RIPA) buffer (Servicebio, China) containing a protease inhibitor cocktail (Sigma, USA). Following quantification by the bicinchoninic acid (BCA) method, the proteins were applied to a 4–20% polyacrylamide gel (GenScript, USA). Following electrophoresis, the separated proteins were transferred to a polyvinylidene fluoride membrane (Millipore, USA), which was then blocked in 5% skim milk at room temperature. The primary antibody (1 : 500) was incubated with the membrane overnight at 4°C. Following three washes in 1 × PBS buffer (pH 7.4), the membrane was treated with the secondary antibody (1 : 2000) for 2 hours at room temperature. The blots were developed using Enhanced Chemiluminescence Kit (FDbio Science, China), and the intensities of protein bands were measured using ImageJ software.

### 2.7. Quantitative Real-Time Polymerase Chain Reaction

Total RNAs were isolated using TRIzol reagent (Invitrogen, USA) and were quantified by the Nano-drop spectrophotometer. The RNA was reverse-transcribed into cDNA by the High-Capacity cDNA Reverse Transcription Kit (Thermo Fisher Scientific, USA). Quantitative real-time polymerase chain reaction (qRT-PCR) was performed with 40 cycles of alternate temperatures of denaturation, annealing, and extension using the SYBR Green PCR Master Mix (Thermo Fisher Scientific, USA). The relative gene expression levels were calculated by the 2^−*ΔΔ*Ct^ method with GAPDH as the reference gene for LINC01614 and HMGA1 and U6 as the reference gene for miR-217-5p. All the experiments were performed in triplicate.

### 2.8. Subcellular Fractionation

First, cytoplasmic and nuclear RNAs were isolated by the PARIS Kit (Life Technologies, MA, USA). Then, qRT-PCR was performed to quantify LINC01614 in cytoplasm and nucleus using *GAPDH* and *U6* as respective references.

### 2.9. Luciferase Reporter Gene Assay

Approximately 5 × 10^4^ cells were seeded into a 96-well plate followed by incubation for 24 hours. After transfection/co-transfection for 48 hours with Lipofectamine 2000 (Invitrogen, USA), luciferase activity was measured using a Luciferase assay kit (Promega, USA) according to the provided protocol. In short, 100 *μ*L of luciferase assay solution was added to an equal volume of cell suspension followed by incubation for 20 minutes at room temperature. Luciferase activity was determined using the Synergy H4 Hybrid Reader (BioTek, Winooski, USA) [17].

### 2.10. RNA Pull-Down and RNA Immunoprecipitation Assay

RNA pull-down protocol was previously described [18]. Biotin-labeled bio-LINC01614 probes were provided by Sangon Biotech (Shanghai, China). The cells were lysed, and one-half of the lysate was used for input control, while the other half was incubated with magnetic Dynabeads M-280 Streptavidin beads (Invitrogen, USA) overnight at 4°C. Finally, miR-217-5p enrichment was determined by qRT-PCR assay.

RNA immunoprecipitation (RIP) was performed using the Magna RNA Immunoprecipitation (RIP) Kit (Millipore, Bedford, MA) according to the manufacturer's guidelines. Briefly, cell lysates were fully mixed with Sepharose beads coupled with the AgO_2_-specific antibody (Cell Signaling Technology, USA) at 4°C for 4 hours. IgG antibody was used as the control. Finally, RNA was extracted from the beads followed by qRT-PCR to determine the relative enrichment [19].

### 2.11. Transwell Assay

The migration and invasion potential of differently treated CRC cells were examined by the transwell assay as per previously described procedures [20]. For invasion assay, the cells were suspended in a serum-free cell culture medium in an upper chamber with a porous membrane containing Matrigel solution (BD, USA). The lower chamber was immersed in a solution containing a complete cell growth medium. Following incubation for 24 hours at 37°C, the cells at the upper side of the porous membrane were gently removed, while the cells retained at the lower side were collected. The collected cells were fixed with 4% formaldehyde solution, stained with 0.1% crystal violet solution, and counted under a light microscope (Olympus, Japan). Migration analysis was performed using the same protocol without the usage of Matrigel.

### 2.12. Cell Cycle Analysis

The cell suspension was digested after 72 hours of transfection. Afterward, the cells were fixed with ethanol (75%) for 4 hours at 4°C, and the supernatant was then discarded, followed by incubation with an RNA enzyme containing iodide (PI, 40%, Sigma). After washed with PBS three times, the cell cycle was detected by using FACS Calibur (BD Biosciences, USA).

### 2.13. Xenograft Tumorigenesis

Nude mice were obtained from the Shanghai Animal Research Center (Shanghai, China). The animal experiment was approved by the Ethics Committee of Yantai Yuhuangding Hospital. sh-NC- and sh-LINC01614#1-transfected CRC cell line (HT-29) (1 × 10^6^ cells/mice) were injected subcutaneously into the mice. Tumor volume was measured weekly. After 5 weeks, the mice were euthanized, and tumor weight and volume were measured.

### 2.14. Statistical Analysis

Statistical analysis was conducted by GraphPad Prism software version 6. Kaplan–Meier analysis was used to compare the survival difference between patient groups. Student's *t* test was used for comparisons between two groups, while one-way ANOVA was used for the statistical analysis of multiple groups. *P* values less than 0.05 were considered significant. The data were presented as the mean ± SD. Each experiment was repeated at least three times.

## 3. Results

### 3.1. LINC01614 Is Upregulated in CRC Tissues and Cell Lines

**Figure 1:**
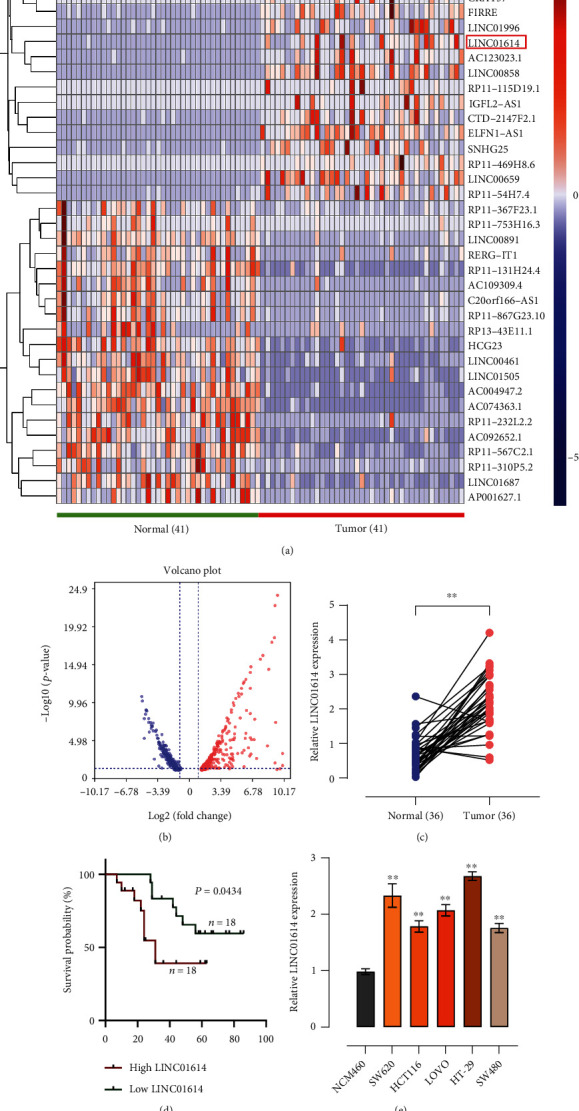
LINC01614 is significantly upregulated in CRC and is associated with poor prognosis. (a) Differentially expressed genes in CRC and paracancerous tissues in the TCGA database. (b) Volcano plot revealing the differential expression of LINC01614 in the CRC tissues in the GEO database. (c) qRT-PCR detecting LINC01614 expression in 36 pairs of CRC and matched normal tissues. (d) Kaplan–Meier analysis analyzing the association between the expression of LINC01614 and the overall survival of CRC patients. (e) qRT-PCR determining LINC01614 expression in the normal colon epithelial line, NCM460, and the five CRC cell lines. Data are presented as the mean ± SD. ∗∗*P* < 0.01.

First, we analyzed LINC01614 expression in 41 pairs of cancer and normal tissues in the TCGA database and found that LINC01614 was upregulated in cancer tissues (Figures 1(a) and 1(b)). To confirm this, we performed qRT-PCR to quantify LINC01614 abundance in 36 clinical samples of CRC. LINC01614 expression was significantly higher (*P* < 0.01) in the CRC than in the control tissues (Figure 1(c)). Then we investigated the correlation of LINC01614 expression levels with the survival of the patients with CRC. We divided the 36 patients into a high expression group (*n* = 18) and a local expression group (*n* = 18) according to the median expression level of LINC01614. Kaplan–Meier survival analysis indicated that CRC patients with high expression of LINC01614 had low survival rates and poor prognosis (*P* = 0.0434) (Figure 1(d)). The high expression group had significantly bigger tumor size, greater Tumor Node Metastasis (TNM) stages, and higher incidences of lymph node and distant metastasis than the low expression group (*P* < 0.01), and age and gender were not significantly different between the two groups (Table 1). Moreover, we determined LINC01614 expression in the CRC cell lines (SW620, HCT116, LOVO, HT-29, and SW480) and the normal colon epithelial cell NCM460 using qRT-PCR. We found that LINC01614 expression was significantly (*P* < 0.01) upregulated in CRC cell lines, a result that was in line with LINC01614 expression in cancer tissues (Figure 1(e)).

### 3.2. LINC01614 Knockdown Inhibits CRC Cell Proliferation and Invasion

**Figure 2:**
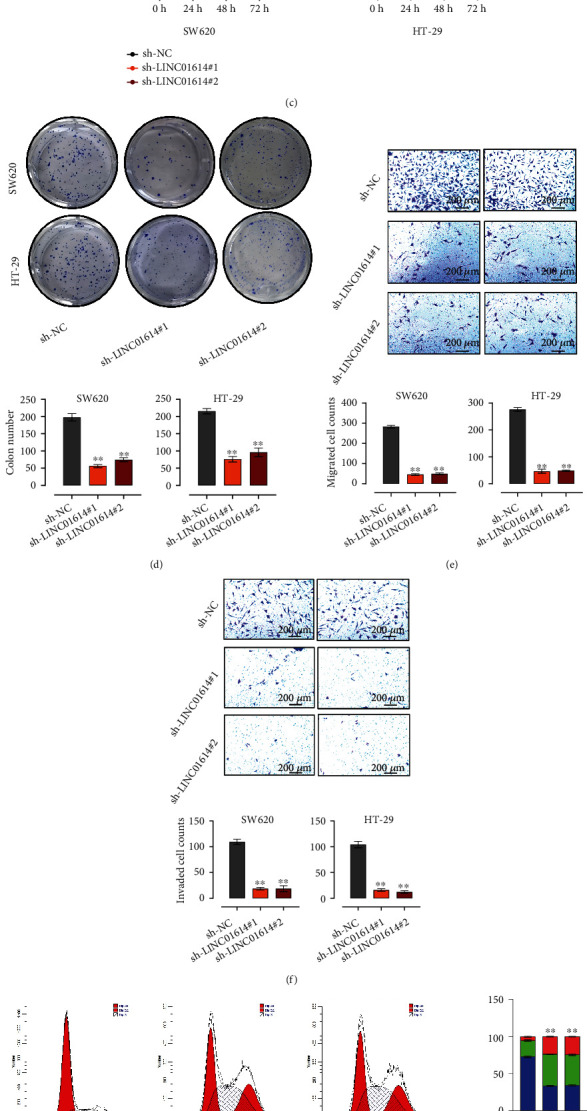
Downregulation of LINC01614 inhibits CRC cell proliferation, migration, and invasion. (a) qRT-PCR revealing the subcellular location of LINC01614. (b) qRT-PCR measuring LINC01614 in HT29 and SW620 cells transfected with sh-NC, sh-LINC01614#1, or sh-LINC01614#2. (c) CCK8 assay examining the proliferation of HT29 and SW620 cells transfected with sh-NC, sh-LINC01614#1, or sh-LINC01614#2. (d) Colony formation assay detecting the cell growth of HT29 and SW620 cells transfected with sh-NC, sh-LINC01614#1, or sh-LINC01614#2. (e and f) Transwell assay checking the migration and invasion of HT29 and SW620 cells after LINC01614 downregulation. (g) Cell cycle was verified by cell cycle assays in HT29 and SW620 cell lines after LINC01614 downregulation. Data are presented as the mean ± SD. ∗∗*P* < 0.01.

To explore the function of LINC01614, we first defined the subcellular localization of LINC01614 in the SW620 and HT-29 cells that exhibit the highest expression levels of LINC01614. Using qRT-PCR, we detected that LINC01614 was mostly enriched in the cytoplasm (Figure 2(a)). Further, we designed two shRNAs targeting LINC01614 (sh-LINC01614#1 and #2) to knock down LINC01614 expression in the SW620 and HT-29 cell lines (Figure 1(e)). QRT-PCR revealed that the knockdown efficiency of both shRNAs was more than 50% (Figure 2(b)). LINC01614 knockdown decreased CRC cell viability, which was manifested by decreased absorption values at 450 nm after 0, 24, 48, and 72 hours of treatment (Figure 2(c)). In addition, the colony formation assay revealed that LINC01614 knockdown decreased the proliferation of CRC cells (Figure 2(d)). Moreover, using the flow cytometry analysis, we found that LINC01614 knockdown enhanced the apoptosis of SW620 and HT-29 cells (Figure S1(a)). Furthermore, LINC01614 silencing significantly decreased the migration (Figure 2(e)) and invasion of SW620 and HT-29 cells (*P* < 0.01) (Figure 2(f)). In order to further explore the role of LINC01614 in CRC progression, cell cycle was investigated after LINC01614 knockdown *in vitro*. The cell cycle analysis showed that more CRC cells were distributed in G1 phase and less in S phase after silencing LINC01614, which suggested that CRC cells were arrested at G1 phase by sh-LINC01614#1/2 (Figure 2(g)).

### 3.3. LINC01614 Sponges miR-217-5p in Colorectal Cancer

**Figure 3:**
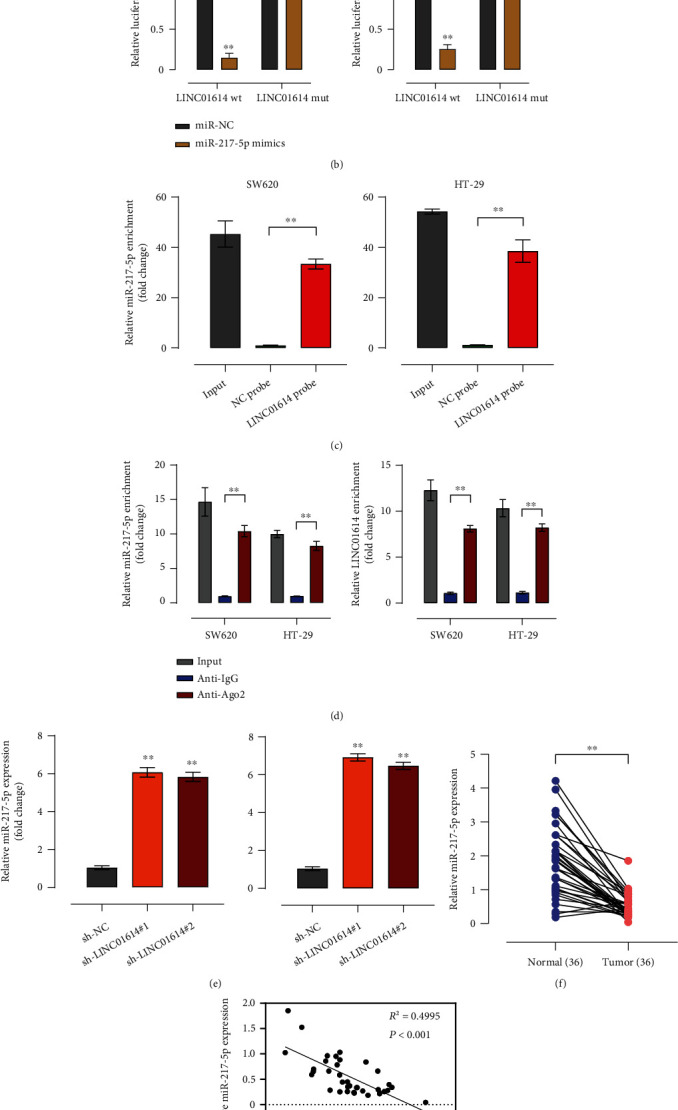
LINC01614 sponges miR-217-5p in CRC. (a) A predicted binding site of miR-217-5p within LINC01614 by LncBase. (b) Luciferase reporter assay evaluating the activity of LINC01614 wt/mut in HT29 and SW620 cells transfected with the negative control (miR-NC) or miR-217-5p mimics. (c) RNA pull-down assay measuring miR-217-5p enrichment in LINC01614 probe. (d) RNA immunoprecipitation (RIP) assays detecting the interaction between LINC01614 and miR-217-5p in HT29 and SW620 cells. (e) qRT-PCR detecting the relative miR-217-5p expression in HT29 and SW620 cells transfected with sh-NC, sh-LINC01614#1, or sh-LINC01614#2. (f) Relative miR-217-5p mRNA expression in 36 pairs of CRC tissues and matched normal tissues. (g) Pearson's correlation coefficients detecting the correlation between LINC01614 and miR-217-5p in CRC tissues. Data are presented as the mean ± SD. ∗∗*P* < 0.01.

Next, we sought to identify the target of LINC01614. Using LncBase v.2, we predicted that LINC01614 contained a potential binding site for miR-217-5p (Figure 3(a)). We first used qRT-PCR to detect the expression of miR-217-5p in both SW620 and HT-29 cells transfected with miR-217-5p mimics (Figure S1(d)). The results have shown that in cells transfected with miR-217-5p mimics, the level of miR-217-5p increased significantly than the control. We then performed the luciferase reporter gene assay in SW620 and HT-29 cells and found that miR-217-5p mimics decreased luciferase activity (Figure 3(b)); however, the mutant of the putative binding site did not (Figure 3(b)). The RNA pull-down assay indicated that LINC01614 probe enriched greater proportion of miR-217-5p in SW620 and HT-29 cells (*P* < 0.01) (Figure 3(c)). Moreover, anti-Ago2 enriched greater amounts of miR-217-5p than did Anti-IgG (*P* < 0.01) (Figure 3(d)). Furthermore, LINC01614 knockdown significantly increased miR-217-5p expression in SW620 and HT-29 cells (*P* < 0.01) (Figure 3(e)). Interestingly, CRC tissues exhibited significantly lower miR-217-5p levels (Figure 3(f)). In addition, LINC01614 expression levels were inversely correlated with miR-127-5p expression levels (*R*^2^ = 0.4995, *P* < 0.001) (Figure 3(g)).

### 3.4. miR-217-5p Targets HMGA1 in Colorectal Cancer

**Figure 4:**
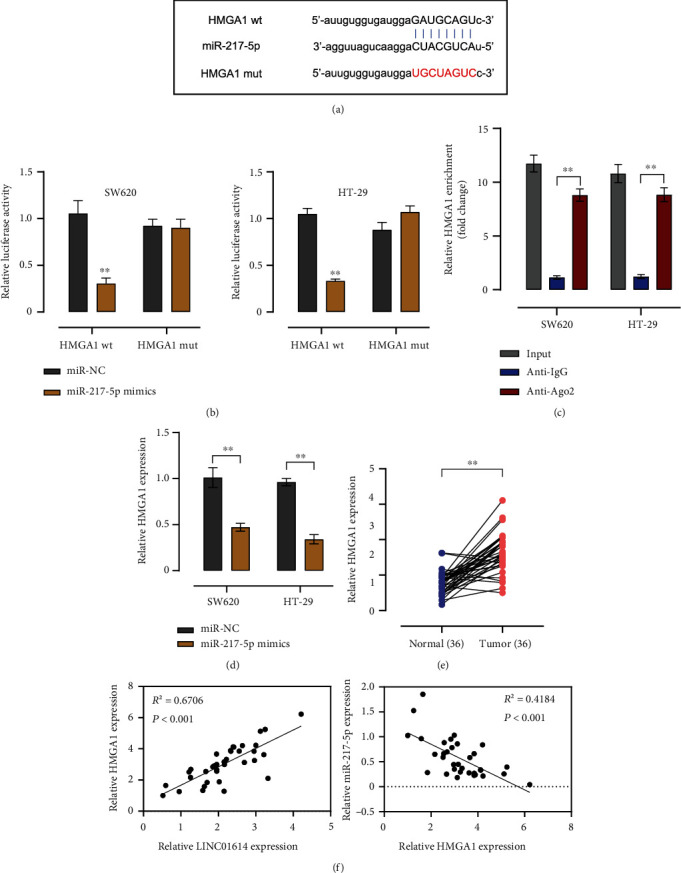
miR-217-5p directly binds to HMGA1. (a) A putative binding site of HMGA1 within miR-217-5p predicted by starBase. (b) Luciferase activity of HMGA1 wt/mut in HT29 and SW620 cells transfected with miR-NC or miR-217-5p mimics. (c) RIP assays detecting the interaction between HMGA1 and miR-217-5p in HT29 and SW620 cells. (d) qRT-PCR measurements to check HMGA1 levels in HT29 and SW620 cells transfected with miR-NC or miR-217-5p mimics. (e) Relative HMGA1 mRNA levels in 36 pairs of CRC tissues and matched normal tissues. (f) Pearson's correlation coefficients detecting the correlation among LINC01614, miR-217-5p, and HMGA1 in CRC tissues. Data are presented as the mean ± SD. ∗∗*P* < 0.01.

StarBASE predicted that miR-217-5p contains a binding site for HMGA1 (Figure 4(a)). Therefore, we reasoned that miR-217-5p could target HMGA1. To test this hypothesis, we performed the luciferase reporter gene assay. We observed that miR-217-5p mimics significantly inhibited the luciferase activity in SW620 and HT-29 cells (*P* < 0.01), an effect that was abrogated by mutating this binding site (Figure 4(b)). Moreover, anti-Ago2 enriched greater amounts of HMGA1 than did Anti-IgG (*P* < 0.01) (Figure 4(c)). In addition, miR-217-5p mimics decreased HMGA1 expression in SW620 and HT-29 cells (Figure 4(d)). qRT-PCR analysis indicated that HMGA1 is significantly higher in CRC than in the normal tissues (Figure 4(e)). Moreover, we observed a positive correlation (*R*^2^ = 0.6706, *P* < 0.001) between the expression levels of HMGA1 and LINC01614, as well an inverse correlation between the expression levels of miR-217-5p and HMGA1 (*R*^2^ = 0.4184, *P* < 0.001) (Figure 4(f)).

### 3.5. LINC01614 Promotes CRC Cell Metastasis by Up-Regulating HMGA1 via Sponging miR-217-5p

**Figure 5:**
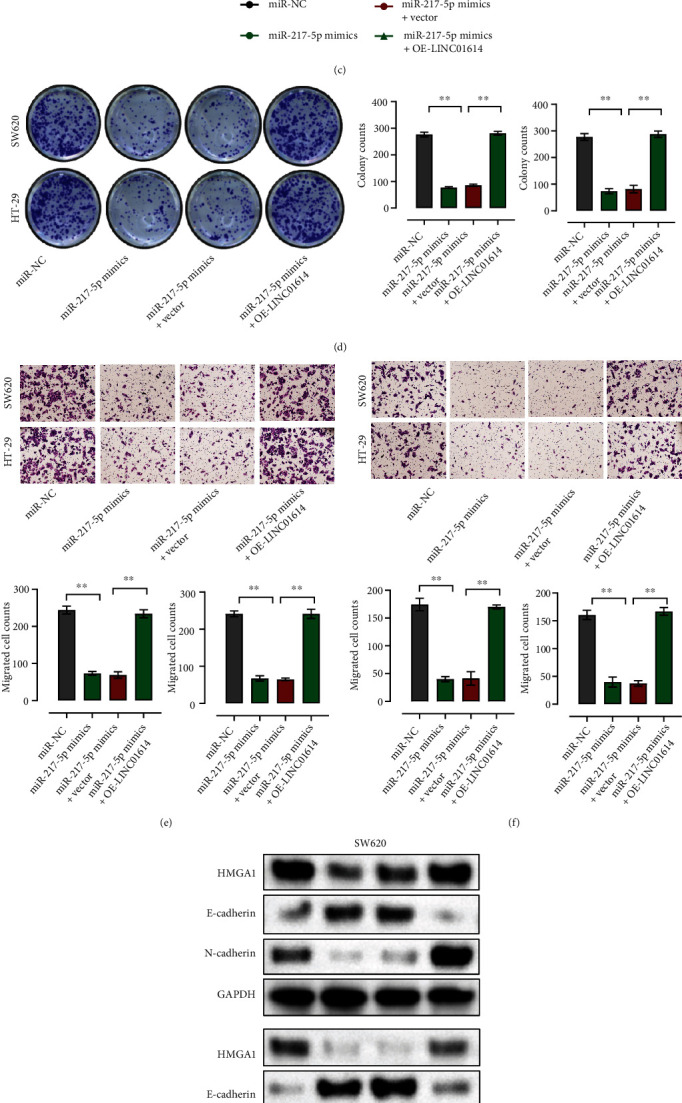
LINC01614 promotes CRC progression through the miR-217-5p/HMGA1 axis. (a) qRT-PCR measurements to determine the LINC01614 level in HT29 and SW620 cells transfected with empty vector or OE-LINC01614. (b) mRNA levels of HMGA1 in HT29 and SW620 cells transfected with miR-NC, miR-217-5p mimics, or miR-217-5p mimics+OE-LINC01614. (c and d) CCK8 and colony formation assay evaluating the cell proliferation of HT29 and SW620 cells transfected with miR-NC, miR-217-5p mimics, or miR-217-5p mimics+OE-LINC01614. (e and f) Transwell assay examining the migration and invasion of HT29 and SW620 cells transfected with miR-NC, miR-217-5p mimics, or miR-217-5p mimics + OE-LINC01614. (g) Western blot analysis detecting the effect of miR-NC, miR-217-5p mimics, and miR-217-5p mimics+OE-LINC01614 on the expression of HMGA1, E-cadherin, and N-cadherin. (h) Cell cycle was verified by cell cycle assays in HT29 and SW620 cells transfected with miR-NC, miR-217-5p mimics, or miR-217-5p mimics+OE-LINC01614. Data are presented as the mean ± SD. ∗∗*P* < 0.01.

To study how LINC01614 affects CRC progression, we overexpressed LINC01614 in SW620 and HT-29 cells through transfection with OE-LINC01614. qRT-PCR measurements confirmed the efficiency of transfection and overexpression (Figure 5(a)). miR-217-5p overexpression effectively decreased HMGA1 expression (*P* < 0.01), which was partially restored by overexpressing LINC01614 (Figure 5(b)). The cell viability (Figure 5(c)), proliferation (Figure 5(d)), migration (Figure 5(e)), and invasion capacity (Figure 5(f)) of CRC significantly became weaker after transfection with miR-217-5p-mimics, which was partially restored by LINC01614 overexpression. Interestingly, transfection of miR-217-5p-mimics decreased the protein levels of HMGA1 and N-cadherin, while increasing the protein level of E-cadherin in HT-29 and SW620 cells (Figure 5(g)). In contrast, LINC01614 overexpression partially reversed the effect of miR-217-5p mimics (Figure 5(g)). The cell cycle analysis showed that more CRC cells were distributed in G1 phase and less in S phase after overexpressing miR-217-5p, in contrast, LINC01614 overexpression partially reversed the effect of miR-217-5p mimics (Figure 5(h)).

### 3.6. LINC01614 Knockdown Inhibits CRC Tumor Growth *In Vivo*

To examine how LINC01614 affects tumor growth *in vivo*, we established a tumor xenograft model in nude mice. Because the knockdown efficiency of sh-LINC01614#1 and sh-LINC01614#2 and the cell phenotype they caused were similar, we just chose the cells transfected with sh-LINC01614#1 for *in vivo* experiments. HT-29 cells were stably transfected with sh-NC and sh-LINC01614#1 and were injected subcutaneously into nude mice. Tumor volume detection revealed a decrease in subcutaneous tumor volume in the sh-LINC01614#1 group compared to the sh-NC group (Figure S1(b)). Similarly, we found that LINC01614 knockdown significantly reduced CRC tumor weight (Figure S1(c)). Collectively, the above data suggest that LINC01614 knockdown inhibits CRC tumor growth *in vivo*.

## 4. Discussion

LncRNAs typically regulate gene expression through sponging miRNAs [21]. Here, using a combination of bioinformatics prediction, luciferase reporter gene assay, RIP, and RNA pull-down assay, we demonstrated that LINC01614 directly targeted miR-217-5p. miR-217-5p largely acts as a tumor suppressor in cancer pathogenesis. For example, bladder cancer tissue-derived exosomes suppress the ferroptosis of T24 bladder cancer cells by transporting miR-217 [22]. miR-217 downregulation predicts poor prognosis in acute myeloid leukemia [23]. In the current study, miR-217-5p mimics decreased the proliferation, migration, and invasion capacity of CRC cells. Similarly, in hepatocellular carcinoma, miR-217-5p suppresses cell invasion [24]. In CRC, miR-217-5p induces cell apoptosis [25]. It has been reported that miR-217 regulates CRC progression through the mitogen-activated protein kinases (MAPK) signaling pathway [26]. Moreover, miR-217 inhibits the proliferation and invasion of CRC cells through an AEG-1-dependent mechanism [27]. In sum, our work together with others strongly suggests that miR-217-5p acts as a tumor suppressor.

Our results indicated that HMGA1 was the target of miR-217-5p. Low levels of HMGA1 were correlated with weak cell proliferation and invasion, while high levels of HMGA1 were correlated with poor patient survival. HMGA1 was first discovered in aggressive cervical cancer cells [28] and is involved in the pathogenesis of various malignant cancers [29]. Increasing studies have shown that HMGA1 expression is elevated in malignant cancers including hepatocellular carcinoma [30], breast cancer [31, 32], gastric cancer [33], and uterine cancer [34], as well as glioblastoma [35], and osteosarcoma [36]. HMGA1 was found to be upregulated in CRC [37, 38], which corroborates our finding that HMGA1 is dysregulated in CRC. The decrease in HMGA1 expression caused by miR-217 overexpression was associated with decreased N-cadherin expression and increased E-cadherin expression in CRC cells. E/N-cadherin has been suggested as a toggle to regulate tumor progression [39]. The decreased expression of E-cadherin has been reported to be associated with poor prognosis in CRC [40] and the invasiveness of colon cancer [41]. Hence, we can conclude that E-cadherin, whose expression is regulated by miRNAs/HMGA1 axis, plays an inhibitory role in CRC progression.

In conclusion, our results suggest that LINC01614 promotes CRC progression through the HMGA1/miR-217-5p axis, potentiating it as a prognostic biomarker for CRC.

## Figures and Tables

**Table 1:** Correlation between LINC01614 expression and clinicopathological variables of CRC patients.

	Total	LINC01614		*P* value
		High (18)	Low (18)	
Age				
<60	21	10	11	0.735
≥60	15	8	7	
Gender				
Male	17	8	9	0.738
Female	19	10	9	
Tumor size				
<5 cm	14	3	11	0.006∗
≥5 cm	22	15	7	
Lymph node metastasis				
NO	19	5	14	0.003∗
YES	17	13	4	
TNM stage				
I + II	17	5	12	0.019∗
III + IV	19	13	6	
Distant metastasis				
No	21	7	14	0.018∗
Yes	15	11	4	
Histological differentiation				
Well moderate	22	6	16	0.001∗
Poor	14	12	2	

p < 0.05.

## Data Availability

The data are available from the corresponding author upon reasonable request.

## Supplementary Materials

Supplementary Materials Knockdown of LINC01614 inhibits CRC tumor growth *in vivo*.
