# Supplementary material for: LINC01614 Promotes Colorectal Cancer Cell Growth and Migration by Regulating miR-217-5p/HMGA1 Axis
Source: Anal Cell Pathol (Amst). 2023 May 31;2023:6833987. doi: 10.1155/2023/6833987 (PMC11401691; doi:10.1155/2023/6833987)
Supplement: Supplementary Materials — Knockdown of LINC01614 inhibits CRC tumor growth in vivo. [file 6833987.f1.docx]

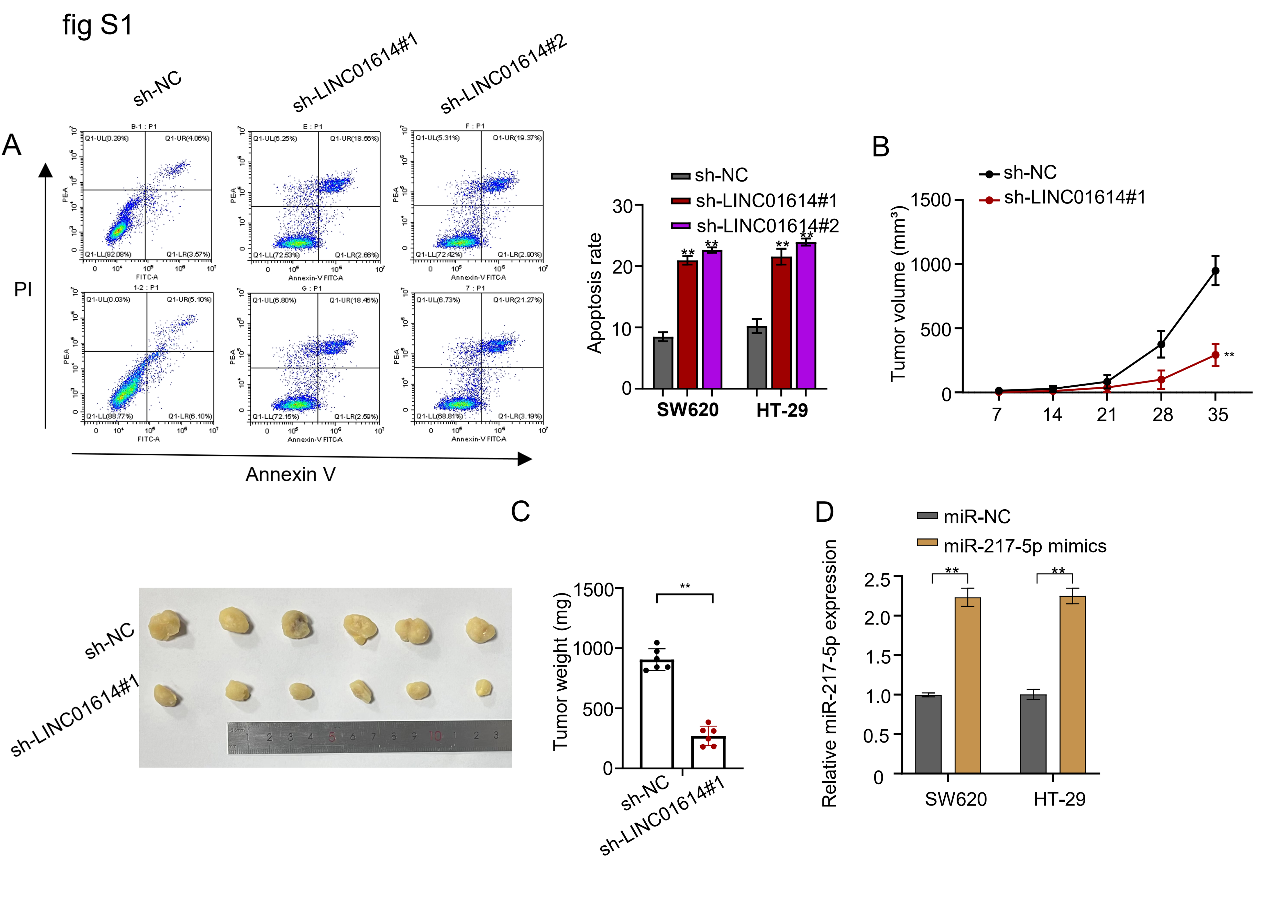


**Figure S1. Knockdown of LINC01614 inhibits CRC tumor growth *in vivo*.**

(A) Numbers of apoptotic cells transfected with sh-LINC01614 calculated by flow cytometry analysis.

(B) Tumor volume in the mice subcutaneously injected with the CRC cells transfected with sh-NC or sh-LINC01614#1.

(C) Tumor weight in the mice subcutaneously injected with the CRC cells transfected with sh-NC or sh-LINC01614#1.

(D) QRT-PCR detected the expression level of miR-217-5p in HT29 and SW620 cells transfected with miR-217-5p mimics.

Data are presented as the mean ± SD. ***P* < 0.01. n=12.
